# Saturated fats network identified using Gaussian graphical models is associated with metabolic syndrome in a sample of Iranian adults

**DOI:** 10.1186/s13098-022-00894-x

**Published:** 2022-08-26

**Authors:** Reihaneh Jahanmiri, Kurosh Djafarian, Nasim Janbozorgi, Fatemeh Dehghani-Firouzabadi, Sakineh Shab-Bidar

**Affiliations:** 1 Department of Community Nutrition, School of Nutritional Sciences and Dietetics, Tehran University of Medical Sciences (TUMS), No 44, Hojjat-dost Alley, Naderi St., Keshavarz Blvd, Tehran, Iran; 2 Department of Clinical Nutrition, School of Nutritional Sciences and Dietetics, Tehran University of Medical Sciences, Tehran, Iran

**Keywords:** Gaussian graphical models, GGMs, Dietary patterns, Dietary networks, Metabolic syndrome

## Abstract

**Background:**

Gaussian graphical models (GGM) are an innovative method for deriving dietary networks which reflect dietary intake patterns and demonstrate how food groups are consuming in relation to each other, independently. The aim of this study was to derive dietary networks and assess their association with metabolic syndrome in a sample of the Iranian population.

**Methods:**

In this cross-sectional study, 850 apparently healthy adults were selected from referral health care centers. 168 food items food frequency questionnaire was used to assess dietary intakes. Food networks were driven by applying GGM to 40 food groups. Metabolic syndrome was defined based on the guidelines of the National Cholesterol Education Program Adult Treatment Panel III (ATP III).

**Results:**

Three GGM networks were identified: healthy, unhealthy and saturated fats. Results showed that adherence to saturated fats networks with the centrality of butter, was associated with higher odds of having metabolic syndrome after adjusting for potential confounders (OR = 1.81, 95% CI 1.61–2.82; P trend = 0.009) and higher odds of having hyperglycemia (P trend = 0.04). No significant association was observed between healthy and unhealthy dietary networks with metabolic syndrome, hypertension, hypertriglyceridemia and central obesity. Furthermore, metabolic syndrome components were not related to the identified networks.

**Conclusion:**

Our findings suggested that greater adherence to the saturated fats network is associated with higher odds of having metabolic syndrome in Iranians. These findings highlight the effect of dietary intake patterns with metabolic syndrome.

## Introduction

Metabolic syndrome (MetS) is a group of interrelated metabolic disorders that makes a person high risk for cardiovascular disease (CVD) and type 2 diabetes (T2D) and therefore it is associated with mortality and morbidity [1]. MetS is defined as a cluster of risk factors including glucose intolerance (type 2 diabetes, impaired glucose tolerance, or impaired fasting plasma glucose), insulin resistance, abdominal obesity, dyslipidemia, and elevated blood pressure (BP) [2–4]. This syndrome and its relevant chronic diseases are a major worldwide public health concern [5]. The prevalence of this syndrome is estimated about 3.3% in all people, 11.9% in obese children and 29.2% in obese population [6]. The worldwide growing prevalence of this syndrome has shown that the estimation of this syndrome is about 10–30% in Asia. The prevalence of this syndrome is about 36.9% in Iran which is higher than many countries in the world [1].

Various research groups have shown that diet is one of the major factors in developing MetS. In recent decades, some studies have investigated the association between MetS and intake of specific food groups, individual foods and nutrients [7, 8]. Furthermore, food and nutrients are consuming in many different combinations. Therefore, dietary pattern analysis has come out as an alternative and completing method to show the complex relationship between dietary intake and risk of chronic disease [9]. Till now, many studies have investigated the association between dietary patterns and the risk of MetS all over the world. Several studies investigate the association between diet and MetS in different societies such as Iran, Thailand, and china [1, 10, 11]. Diet-disease studies usually use dietary pattern which derive by using data reduction techniques like principal component analysis (PCA) or cluster analysis [12–15]. In dietary patterns, each food group can be related to more than one pattern and the pairwise correlation between groups are dependent of the effect of other food groups. Gaussian Graphical models (GGM) were recently introduced as a commanding method for dietary pattern analysis which shows conditional independencies between food groups [16]. This analysis was emerged as a comprehensive alternative or supplemental method to understanding diet–disease relationship [17–19]. A few studies have been addressed this method to find diet-disease relationships. Iqbal et al. have identified networks of dietary intake in German adult population in 2016. They have found a major network in men and also women which consist of red and processed meat, poultry, cooked vegetables, sauces potatoes, cabbage, mushrooms, legumes, soup, whole grain and refined bread. However, in women, it also consists of fried potatoes [16]. In 2018, Iqbal et al. have investigated the association between the identified networks and risk of chronic diseases. They found that higher adherence to western-type pattern was associated with higher risk of type 2 diabetes (T2DM) in women. It is worthy to mention that adherence to a high-fat dairy pattern causes low risk of T2DM in men and women [17]. Existence of different cultures and way of life in Iran leads to various dietary patterns and habits. Then, the aim of this study is to explore dietary networks to understand which food groups have centrality and then evaluate its association with metabolic syndrome.

## Methods

### Subjects

In this cross-sectional, we recruited 850 adults, of whom 69% were women with age range of 20–59 years and lived in Tehran, Iran, using two-stage cluster random sampling from 2018 to 2019. The sampling was conducted by dividing the health centers of Tehran into five regions: north, south, east, west and center. Then, randomly selected participants, equitably sampled from the five regions, were recruited from 25 health centers (according to budgetary and time constraints). Subjects were considered eligible for inclusion if the following criteria were met: (a) participants within the age range of 20–59 years; (b) apparently healthy individuals who did not report any previous diagnosis of chronic diseases such as diabetes, cardiovascular diseases and chronic kidney, lung and liver diseases by a physician; (c) be willing to take part in study; (d) being a resident of Tehran; and (e) being a member of the health center. Participants were excluded from the analysis if: (a) their daily energy intake was implausibly low or high (< 800 kcal/day or > 4200 kcal/day); and (b) those who did not report any adherence to certain dietary patterns, any special diet or diet therapy such as vegetarian diet. The exclusion criteria were pregnancy and lactation, kidney, liver or long diseases, cancer, myocardial infarction and any kind of disease which can affect diet or be an obstacle for blood tests.

### Ethical statement

All procedures were in accord with the ethical standards of the Tehran University of Medical Sciences (IR.TUMS.MEDICINE.REC.1399.1104), who approved the protocol and informed consent form. All participants signed a written informed consent prior to the start of the study.

### Data collection

Data were collected using an interview by trained staffs. Demographic data including age, sex, education, job, smoking status and marital status were collected by questionnaire. Education level was categorized as under diploma, diploma and graduated. Marital status and job sorts were classified to single, married and other cases, and working or not working, respectively. Physical activity information was collected using international physical activity questionnaire (IPAQ). This activity was categorized according to minutes per week into three groups as light, moderate and heavy physical activity. Usual dietary intake was assessed by valid, reliable, semi-quantitative and 168 items food frequency questionnaire (FFQ). Trained nutrition experts have asked participants about food frequency intake and their portion size in face-to-face interviews. Ultimately, food consumption quantity was considered to gram per day for each person. Results were entered to the excel file which was designed for calculating weight of food in gram. Amount of standard units and the items which were reported as household measures were converted to gram using household measures guide. Finally, the equivalent consumption of each food items for each person in gram was obtained. Energy of the food items was determined using USDA food composition database. Iranian food composition table was used for some items which could not be found in USDA database. Anthropometric indicators including height, weight, waist circumference and hip circumference were assessed and body mass index (BMI) was calculated using person's weight in kilograms divided by the square of height in meters. Participant’s height was measured with stadiometer without shoes and to the nearest 1 mm.

### Laboratory measurements

Blood pressure was measured in sitting position after 10–15 min’ rest in two phases with at least 30 s interval and the average was reported. 10 cc of blood was taken in fasting situation between 7 to 10 am and poured in acid-washed test tubes without coagulant. Clot was made after 30 min being in room temperature and then was centrifuged with the speed of 3000*g*$\times$ for 20 min. Serums were kept in clean micro tubes in refrigerator at − 80 °C until the end of the experiment. Fasting blood sugar (FBS) test was done using commercial kit (Pars Azmun, Tehran, Iran) with enzymatic spectrophotometric method (glucose oxidase) by automatic device (Selecta E, Vitalab, Netherland). High density lipoprotein (HDL) was measured by phenol Amino Antipyrine cholesterol oxidase method and triglyceride (TG) was also evaluated by glycerol-3 phosphate oxidase phenol amino antipyrine enzymatic method.

### Metabolic syndrome

MetS was diagnosed if the patient had three or more of the following risk factors as established by NCEP-ATP III: large waist circumference (WC > 102 in men and WC > 88 in women), high blood pressure (BP > 130/80), high triglyceride (TG > 150), high glucose (FBG > 110), and low HDL (HDL < 40 in men and HDL < 50 in women) [20].

### Assessment of dietary networks by GGMs

The GGMs method to derive dietary networks was explained before [18]. Briefly, we classified food items recorded by FFQ into 39 food groups (Table 1). The analysis of GGM was performed in R (version 3.4.3, R) [21]. The R package ‘‘glasso’’ was applied to estimate a sparse inverse covariance (precision) matrix [22]. Communities, sets of closely related links, were detected within all identified networks to facilitate interpretation using the R package “linkcomm”, which is able to detect nested and overlapping communities in networks [23].

**Table 1:** Dietary intakes of 39 food groups used to derive dietary networks using GGM

Food groups	Total (n = 850)	Men (n = 266)	Women (n = 584)
	Means ± SD		
Cookies, crackers, cakes	24.8 ± 42.8	27.7 ± 44.3	23.5 ± 42.1
Chips, puffs	9.44 ± 30.3	10.0 ± 35.5	9.17 ± 27.7
Sauce	2.6 ± 3.89	2.22 ± 4.33	1.99 ± 3.76
Processed meat	3.23 ± 7.77	4.8 ± 10.7	2.84 ± 5.96
meat	65.3 ± 75.4	66.1 ± 78.2	65.0 ± 74.2
Carbonated drinks	42.7 ± 120	52.6 ± 135	38.2 ± 113
sweets	32.5 ± 41.5	37.6 ± 47.0	30.2 ± 38.6
spices	38.8 ± 33.7	38.3 ± 33.1	39.8 ± 34.9
dessert	1/01 ± 2.57	1.37 ± 3.01	0.84 ± 2.33
fish	12.2 ± 21.6	11.5 ± 18.6	12.5 ± 22.9
Organ meats	4.97 ± 15.0	5.06 ± 1.20	4.93 ± 12.0
French fries	11.6 ± 36.3	11.9 ± 29.9	11.6 ± 39.6
Fresh fruits	345 ± 405	372 ± 458	332 ± 378
Canned fruits	5.07 ± 27.1	7.72 ± 36.5	3.08 ± 21.1
Fruit juice	28.2 ± 82.8	34.6 ± 92.1	25.3 ± 77.9
Dried fruits	16.1 ± 74.0	20.7 ± 102	13.7 ± 55.6
Cabbage	7.66 ± 23.8	9.71 ± 33.5	6.67 ± 17.1
Garlic	1.15 ± 3.25	1.05 ± 1.93	1.20 ± 3.72
mushroom	5.15 ± 11.7	5.30 ± 9.97	5.08 ± 12.5
Cooked vegetables	99.2 ± 85.2	107 ± 101	95.3 ± 75.6
Green leafy vegetables	31.3 ± 41.1	32.7 ± 46.0	30.7 ± 40.0
Other vegetables	270 ± 250	287 ± 240	264 ± 256
nuts	15.7 ± 33.9	13.2 ± 26.2	16.9 ± 37.0
legumes	34.3 ± 46.0	36.9 ± 48.4	33.1 ± 44.8
High fat dairy	116 ± 202	143 ± 241	103 ± 179
Low fat dairy	354 ± 405	357 ± 373	353 ± 420
Low fat cheese	6.24 ± 1.16	16.9 ± 23.1	16.2 ± 25.3
High fat cheese	6.5 ± 14.3	7.85 ± 14.1	5.93 ± 13.3
Grains	18.0 ± 36.0	20.7 ± 50.6	16.1 ± 26.1
Breads	144 ± 13.8	147 ± 159	143 ± 145
Rice, Pasta, Noodles	273 ± 231	289 ± 255	266 ± 217
Butter	3.50 ± 11.5	4.95 ± 9.36	4.10 ± 15.1
Margarine	2.82 ± 3.11	3.38 ± 11.8	3.00 ± 11.5
Animal fat	2.32 ± 8.17	2.56 ± 10.52	2.20 ± 6.76
Vegetable oils, olive	15.0 ± 24.3	14.3 ± 21.5	15.5 ± 25.5
Cooked potatoes	26.7 ± 31.4	28.7 ± 36.4	25.6 ± 28.6
Egg	21.2 ± 25.1	20.4 ± 21.3	21.6 ± 26.7
Tea	574 ± 707	577 ± 101	572 ± 494
Coffee	26.5 ± 56.7	28.2 ± 57.3	25.7 ± 56.7

Listed are 39 food groups derived from a 168-item FFQ. Independent t test was used to compare mean of dietary intakes between the genders

GGM-derived dietary networks consist of nodes (food groups) and edges (conditional dependencies). Vertices and edges in the networks demonstrated food group(s) and conditional dependencies between them, respectively. Partial correlations ≥ ± 0.30 were considered strong [24] to show conditional dependencies. Positive and negative partial correlations were displayed by solid lines and dashed lines, respectively. The strength of the correlation between foods groups were shown by thickness of edges. A combination of three or more nodes that were related to each other formed a dietary network [25]. Food groups that belonged to more than one community were evaluated for centrality to determine the potential importance of a food group based on the number of communities it belongs to [26]. Central food groups were defined as groups with high correlation by a larger number of other food groups. To compute dietary networks score, dietary intake variables included in each network were standardized to the same mean (i.e., ‘0’) and 1 standard deviation. In the second step, standardized intakes of food groups were multiplied by their factor loading scores (positive or negative) obtained by PCA. Then, the score of food groups within each network were added together to calculate network scores. The network scores were then categorized in tertiles. The first tertile was considered as the reference group.

### Statistical methods

One-way ANOVA and chi-square analysis were used to compare the general characteristics of quantitative and qualitative variables, respectively. Quantitative and qualitative variables were reported as mean ± SD and percentages, respectively and count on P-value less than 0.05.

The odds ratio (ORs) and 95% confidence intervals (CIs) of metabolic syndrome were calculated across tertiles of networks using multiple logistic regression models. OR was determined for three models. Model 1 was crude. Model 2 was adjusted for age, sex, education, occupation, marital status, smoking status, menopause and physical activity. Lastly, model 3 was also adjusted for age, sex, education, occupation, marital status, smoking status, menopause, body mass index and energy intake. P value for trend to show the trend of association across the tertiles was calculated using median values of each tertiles in logistic regression models.

## Results

### General characteristics

Table 2 presents the general characteristics of the study population divided by gender. The proportion of employers was a bit higher in women (51.4%) than men (48.6%) whereas the proportion of retires was higher in men (61.7%) than women (38.3%) (P = 0.001). Men were less married (34.4%, P = 0.001) and more smokers (75%) than women (25%) (P = 0.001). MetS was more prevalent in women (87.6%, P < 0.001).

**Table 2:** General characteristics of study population according to the sex

General characteristic	All(850)	Men(266)	Women(584)	P-value
	Mean ± SD			
Age (year)	44.7(10.8)	45.2 ± 10.1	44.5 ± 11.1	0.39

		n(%)		
Physical activity				0.45
Low	539	162(30.1)	377(69.9)	
Moderate	310	104(34.4)	207(66.6)	
Education				0.057
Under diploma	244	78(34.8)	146(65.2)	
Diploma	261	71(27.2)	190(72.8)	
Graduated	292	101(34.6)	191(65.4)	
Occupation				< 0.001
Employed	220	107(48.6)	113(51.4)	
Household	476	78(16.4)	398(83.6)	
Retired	128	79(61.7)	49(38.3)	
Unemployed	26	2(7/70)	24(92.3)	
Marital Status				< 0.001
Single	92	25(27.2)	67(72.8)	
Married	688	237(34.4)	451(65.6)	
Others	70	4(5.70)	66(94.3)	
Smoking status				
Never smoker	770	216(28.1)	554(71.9)	< 0.001
Ex-smoker	36	17(47.2)	19(52.8)	
Smoker	44	33(75)	11(25)	
Menopause	278	–	278(32.7)	-
Metabolic syndrome				0.15
Yes	250	31(12.4)	219(87.6)	
No	600	235(39.2)	365(60.8)	
Hypertension				0.37
Yes	143	50(35.0)	93(65.0)	
No	707	216(30.6)	491(69.4)	
Hyperglycemia				0.21
Yes	420	129(30.7)	291(69.2)	
No	430	137(31.8)	293(68.3)	
Hypertriglyceridemia				0.52
Yes	320	216(67.5)	104(32.5)	
No	530	162(30.5)	368(69.4)	
Central obesity				0.022
Yes	413	77(18.6)	336(81.3)	
No	437	189(43.2)	248(56.7)	

Chi-square test was used to compare the frequencies between the genders

### Dietary networks

**Fig. 1:**
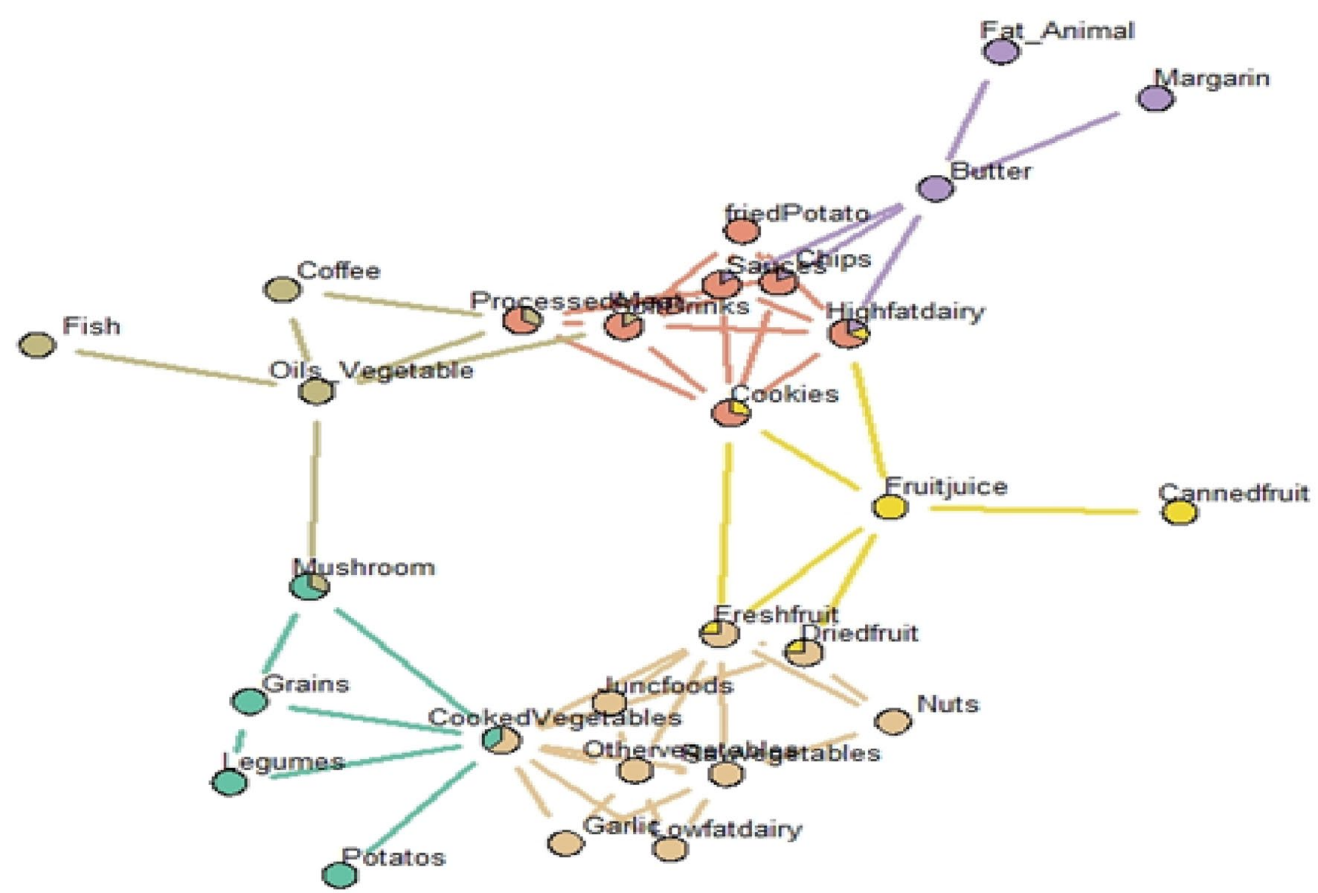
All dietary networks derived by Gaussian graphical models. Vertices and edges in the networks demonstrated food group(s) and conditional dependencies between them respectively. The strength of the correlation between food groups had shown by thickness of edges (n = 850). Reprinted from Dietary networks identified by Gaussian graphical model and general and abdominal obesity in adults, by Jayedi et al. 2021

Three dietary networks were derived by GGMs from the whole study population (Fig. 1). Identified networks were named as healthy, unhealthy and saturated fats. Healthy network consisted of two communities and 13 food groups which cooked vegetables have centrality. The first community consisted of cooked vegetables, mushrooms, grains, legumes and boiled potatoes. Cooked vegetables as a central food group in this network showed an inconsiderable correlation with mushrooms, legumes, boiled potatoes and grains (0.23, 0.20, 0.14, and 0.03, respectively). Grains also had a correlation with mushrooms (0.11) and legumes (0.14). The second community showed the association between consumption of cooked vegetables with fresh fruits (− 0.10), raw vegetables (0.19), garlic (0.11), low fat dairy (0.13), side dish (0.16) and other vegetables (0.16). This community also contained dried fruits and nuts which are inconsiderably correlated to each other (0.16). Nuts also had negative correlation with raw vegetables (− 0.05) and positive one with fresh fruits (0.15). The strongest correlation was seen between fresh fruits and raw vegetables (0.30). Raw vegetables also were correlated with low far dairy (0.07).

Unhealthy network consists of three communities which processed meat plays a central role. The left community shows the consumption of vegetable oils, fish, coffee, processed meat and drinks. Processed meat had correlation with coffee (0.06) and vegetable oils (0.97). Vegetable oils had a negative correlation with Coffee (− 0.05), drinks (− 0.16) and also considerable correlation with fish (0.99). Central community included processed meat, drinks, chips/puff, fried potatoes, high fat dairy, cakes/cookies and Sauce. Processed meat was also correlated with sauce (0.15), drinks (0.20) and cookies/cakes (0.03). Drinks was also with fried potatoes (0.03), cookies/cakes (0.9) and high fat dairy (0.12). Cookies and cakes were also correlated with sauce (0.01), chips/puff (0.06) and high fat dairy (0.11). Fried potatoes had correlation with chips/puffs (0.08) and high fat dairy (0.07). The third community in the right side of the network consisted of fresh fruits, canned fruits, high fat dairy and cookies/cakes which fruit juice showed correlation with canned fruits (0.25) and high fat dairy (0.14) and cookies/cakes (0.18). Saturated fats dietary network consisted of butter, margarine and animal fat. Butter has central role in this network and correlates with animal fat (0.03) and margarine (0.06).

Means and standard deviation (SD) of MetS related biomarkers and anthropometric measurements by tertiles of the three important networks identified by GGM are shown in Table 3. Across the tertiles of healthy network, systolic blood pressure (P = 0.16), high density lipoprotein (P = 0.42) and body weight (P = 0.95) were increased, and triglyceride was decreased (P = 0.14). Throughout the saturated fats network, triglyceride (P = 0.27) and waist circumference were increased (P = 0.40). However, no significant association was seen between MetS components, anthropometric measurements and identified dietary networks. The ORs and 95% CI derived by logistic regression analysis between each dietary networks and MetS, hypertension, hyperglycemia, hypertriglyceridemia and central obesity are shown in Table 4. We found that adherence to saturated fats network is associated with higher odds of MetS when compared with the first tertile after adjusting for potential confounders in model 2 (P = 0.01,95% CI 1.13–2.68 P trend = 0.009) and model 3(P = 0.008, 95% CI 1.16–2.82 P trend = 0.01). However, no significant association was seen between healthy network (P trend for model 3 = 0.92) and unhealthy network (P trend for model 3 = 0.66) with MetS. Saturated fats network was also associated with hyperglycemia after adjusting for potential confounders in model 2 (P trend = 0.04) and model 3 (P trend = 0.05). No meaningful association was seen between tertiles of identified networks with hypertension, hypertriglyceridemia and central obesity.

**Table 3:** Metabolic syndrome related markers by Tertiles (T) of identified dietary networks in study population^a^

	Healthy			P-value^b^	Unhealthy			P-value^b^	Hydrogenated oils			P-value^b^
	T1	T2_2_	T3_3_		T1	T2_2_	T3_3_		T1	T2_2_	T3_3_	
Participants	283	283	283		283	283	283		283	283	283	
Weight(kg)	73.7 ± 13.4	73.5 ± 13.4	73.8 ± 13.8	0.87	72.8 ± 13.6	72.5 ± 12.7	74.9 ± 14.3	0.34	73.5 ± 14.7	73.2 ± 12.9	72.9 ± 12.7	0.61
BMI(kg/m^2)^	27.8 ± 4.90	27.9 ± 6.90	28.7 ± 4.50	0.96	27.8 ± 5.04	28.3 ± 7.20	27.7 ± 4.75	0.23	28.1 ± 7.2	27.6 ± 4.27	27.7 ± 4.68	0.37
WC(cm)	91.4 ± 12.2	92.0 ± 12.0	92.8 ± 12.7	0.39	91.7 ± 12.1	91.9 ± 11.99	92.7 ± 12.8	0.60	91.6 ± 11.8	92.0 ± 11.63	92.7 ± 13.5	0.56
WC/HP	0.88 ± 0.08	0.89 ± 0.16	0.88 ± 0.08	0.27	0.88 ± 0.16	0.89 ± 0.08	0.88 ± 0.08	0.71	0.88 ± 0.16	0.88 ± 0.08	0.89 ± 0.08	0.46
FBS(mg/dl)	105 ± 28.1	108 ± 38.1	108 ± 34.2	0.35	109 ± 39.9	109 ± 57.6	103 ± 23.3	0.09	108 ± 37.4	104 ± 25.6	109 ± 36.8	0.26
TG(mg/dl)	143 ± 76.5	150 ± 82.2	140 ± 71.8	0.25	142 ± 82.8	152 ± 15.0	139 ± 74.5	0.12	137 ± 72.4	146 ± 78.18	149 ± 80.5	0.17
HDL(mg/dl)	49.5 ± 10.1	49.7 ± 10.4	50.3 ± 10.0	0.64	49.5 ± 10.6	49.8 ± 10.0	50.3 ± 9.88	0.67	50.5 ± 10.0	49.1 ± 10.15	49.9 ± 10.4	0.28
SBP(mmHg)	119 ± 18.3	121 ± 16.9	123 ± 19.8	**0.03**	120 ± 18.5	121 ± 17.2	121 ± 19.5	0.85	121 ± 18.1	120 ± 17.5	120 ± 19.6	0.71
DBP(mmHg)	77.9 ± 11.8	79.5 ± 11.4	79.2 ± 11.2	0.20	78.6 ± 11.6	78.5 ± 9.90	79.5 ± 12.8	0.56	78.9 ± 12.2	79.1 ± 11.6	78.5 ± 10.7	0.82

BMI body mass index. WC waist circumference. WC/HP waist circumference to Hip circumference Ratio. FBS fasting blood sugar. TG triglyceride. HDL high density lipoprotein. SBP systolic blood pressure. DBP diastolic blood pressure

^a^Values are presented in means ± SD

^b^p-values were obtained using One way ANOVA

**Table 4:** Tertiles (T) of dietary networks and odds of metabolic syndrome in study population

Exposure	T1		T2		T3			P trend
		OR	95% CI	P	OR	95%CI	P	
Metabolic syndrome								
Healthy								
Model 1	1	0.87	0.6–1.25	0.45	1.03	0.72–1.47	0.85	0.90
Model 2	1	0.87	0.57–1.32	0.77	0.99	0.65–1.51	0.97	0.84
Model 3	1	0.88	0.57–1.35	0.55	1.01	0.66–1.54	0.94	0.92
Unhealthy								
Model 1	1	1.03	0.72–1.48	0.85	0.91	0.63–1.32	0.64	0.64
Model 2	1	0.94	0.62–1.42	0.77	0.91	0.59–1.40	0.67	0.58
Model 3	1	0.93	0.61–1.42	0.74	0.89	0.57–1.37	0.60	0.66
Hydrogenated oils								
Model 1	1	1.41	0.98–2.05	0.96	0.96	0.96–2.01	0.07	0.08
Model 2	1	1.48	0.96–2.28	1.74	1.74	1.13–2.68	0.01	0.01
Model 3	1	1.53	0.99–2.37	1.81	1.81	1.16–2.82	0.008	0.009
Hypertension								
Healthy								
Model 1	1	1.14	0.72–2.80	0.55	1.35	0.87–2.11	0.17	0.17
Model 2	1	1.03	0.56–1.89	0.92	1.40	0.78–2.51	0.25	0.25
Model 3	1	1.03	0.56–1.90	0.90	1.39	0.77–2.48	0.26	0.26
Unhealthy								
Model 1	1	0.87	0.56–1.36	0.55	1.02	0.66–1.58	0.91	0.90
Model 2	1	0.99	0.55–1.76	0.97	1.04	0.56–1.92	0.88	0.90
Model 3	1	0.99	0.55–1.77	0.98	1.04	0.56–1.92	0.88	0.90
Hydrogenated oils								
Model 1	1	1.02	0.65–1.58	0.92	0.95	0.61–1.48	0.82	0.82
Model 2	1	1.11	0.61–2.02	0.72	1.12	0.61–2.05	0.69	0.67
Model 3	1	1.14	0.62–2.07	0.66	1.15	0.63–2.09	0.64	0.63
Hypertriglyceridemia								
Healthy								
Model 1	1	1.15	0.82–1.61	0.41	0.99	0.70–1.39	0.95	0.95
Model 2	1	0.97	0.64–1.45	0.88	0.73	0.48–1.11	0.14	0.15
Model 3	1	0.97	0.65–1.46	0.90	0.73	0.48–1.11	0.15	0.16
Unhealthy								
Model 1	1	1.13	0.81–1.59	0.45	0.94	0.66–1.32	0.72	0.72
Model 2	1	1.17	0.77–1.77	0.44	1.08	0.70–1.65	0.72	0.71
Model 3	1	1.16	0.77–1.76	0.45	1.11	0.72–1.70	0.62	0.61
Hydrogenated oils								
Model 1	1	1.21	0.86–1.70	0.27	1.27	0.901.79	1.64	0.16
Model 2	1	0.91	0.60–1.38	0.66	1.26	0.83–1.92	0.26	0.26
Model 3	1	0.88	0.58–1.35	0.58	1.23	0.81–1.87	0.32	0.32
Hyperglycemia								
Healthy								
Model 1	1	1.16	0.83–1.61	0.37	1.45	1.04–2.02	0.02	0.02
Model 2	1	0.89	0.59–1.34	0.59	1.30	0.86–1.97	0.20	0.22
Model 3	1	0.92	0.61–1.36	0.86	1.31	0.88–1.96	0.18	0.20
Unhealthy								
Model 1	1	1.17	0.84–1.63	0.33	1.00	0.71–1.39	1.00	0.99
Model 2	1	1.16	0.82–1.63	0.39	0.93	0.66–1.32	0.71	0.18
Model 3	1	1.17	0.84–1.63	0.34	0.99	0.71–1.38	0.97	0.32
Hydrogenated Oils								
Model 1	1	1.27	0.91–1.87	0.14	1.34	0.96–1.87	0.08	0.09
Model 2	1	1.34	0.95–1.89	0.09	1.38	0.98–1.95	0.06	0.04
Model 3	1	1.27	0.91–1.78	0.14	1.34	0.96–1.87	0.08	0.05
Central Obesity								
Healthy								
Model 1	1	0.84	0.61–1.18	0.33	1.08	0.77–1.50	0.64	0.64
Model 2	1	0.86	0.54–1.37	0.53	0.89	0.55–1.44	0.66	0.58
Model 3	1	0.86	0.55–1.33	0.50	0.89	0.56–1.39	0.60	0.66
Unhealthy								
Model 1	1	0.93	0.67–1.30	0.70	1.00	0.71–1.39	1.00	0.99
Model 2	1	0.90	0.56–1.44	0.67	0.97	0.60–1.58	0.91	0.86
Model 3	1	0.84	0.54–1.44	0.67	0.97	0.60–1.58	0.91	0.87
Hydrogenated Oils								
Model 1	1	1.11	0.80–1.54	0.52	1.29	0.92–1.79	0.13	0.13
Model 2	1	1.23	0.79–1.93	0.34	1.37	0.87–2.17	0.16	0.16
Model 3	1	1.27	0.79–2.05	0.31	1.39	0.85–2.28	0.18	0.18

Model 1: Crude. model 2: Adjusted for age, sex, education, occupation, menopause, smoking status and activity score. Model 3: Adjusted for age, sex, education, occupation, menopause, smoking status, energy intake and BMI

## Discussion

Overall, in this study we identified three main dietary networks using GGM labelled as healthy, unhealthy and saturated fats. The healthy dietary network consisted of cooked and raw vegetables, fresh and dried fruits, side dish, nuts, garlic, mushroom, grains, legumes, potatoes and other vegetables with the centrality of cooked vegetables. The unhealthy dietary network included processed meat, drinks, sauce, chips/puff, high fat dairy, cookies/cake, fruit juice, canned fruits, coffee, vegetable oils, fried potatoes and fish which processed meat plays a central role. The third identified dietary network is saturated fats which contain butter as a central food group, margarine and animal fat. Following the saturated fats network was associated with greater odds of having MetS and hyperglycemia in the study population after adjusting for potential confounders. We found no association between healthy and unhealthy dietary networks with MetS and its components.

GGM is a probing method for dietary pattern analysis which shows independent correlations between food groups [16] and recently was introduced as a great technique to find diet–disease relationship [17]. Food networks demonstrate how food groups are consuming in relation to each other independently. Central food groups play a key role in comprehending eating behaviors since most of the food groups in a network are consumed in relation to them [16].

To our knowledge, this cross-sectional study is the first study which assesses the association between dietary networks and risk of MetS. The identified networks and their relation with MetS are similar to studies which used PCA method to derive dietary patterns in some populations. A research which was performed in the frame of Isfahan cohort study from 2001 to 2013, has assessed the longitudinal association of dietary patterns and MetS. They found that more adherence to Iranian dietary patterns which consist of high intake of Iranian traditional products like hydrogenated oils, animal fat, dairy products, sweets and organ meat, was associated with higher risk of MetS and number of MetS components [27]. Moreover, some other studies have identified positive association between saturated fats and butter and MetS [28, 29]. Furthermore, in Isfahan cohort study between 2003 and 2008 a direct association was observed between western dietary pattern, which was high in butter and saturated fats, and MetS in impaired glucose tolerance individuals [30, 31]. In another study on Tehrani female teachers, Western diet with high amount of butter and saturated fats was related with higher odds of MetS [23]. Correspondingly, a results of a recent systematic review and meta-analysis on the association of MetS and dietary patterns showed western/unhealthy patterns including butter and hydrogenated fats are associated with increased risk for MetS [32]. Hydrogenated vegetable oils are major source of trans fatty acids (TFA) in both developed and developing countries. TFAs could cause hyperinsulinemia, disturbance of glucose homeostasis and insulin resistance as a result of modifications in the structure of lipids in skeletal muscle and adipose tissue, reduction in activation of protein kinase B in adipose tissue and muscle. High intakes of saturated fats also could cause undesirable modulation in metabolism of lipoprotein, which was illustrated by increase in total and LDL cholesterol, TG, decrease in concentration of HDL cholesterol and apolipoprotein A-I which all related to MetS. Additionally, another mechanism could be inflammatory cytokines production and endothelial dysfunction. It seems that Butter could cause disturbance of lipid profile, increase in waist circumference, body mass index, blood pressure and also triglyceride concentration because of containing high amount of cholesterol and saturated fatty acids [29].

We did not observe any association between healthy and unhealthy dietary networks with MetS and its components. Results of a systematic review and meta-analysis of observational studies have showed that higher adherence to Western/unhealthy dietary pattern was associated with increased risk of MetS, whereas a Healthy/prudent dietary pattern was related to a lower risk of MetS. The Western/unhealthy pattern has included red meat, processed meat, sweets, French fries, desserts, refined grains, eggs and high fat dairy products which was partially similar to our unhealthy network [33].

In a study by Iqbal et al., Western type GGM network was associated with higher risk of type 2 diabetes in women and higher BMI in both sexes [17]. In another study, risk of central obesity for women in fourth quartile of energy and saturated fatty acids intake was higher than for those in the first quartile [34]. A three years cohort study also shows saturated fats and butter consumption was positively associated with changes of waist circumference, fasting blood sugar and triglyceride concentration [29]. Considerably, our unhealthy network contains some food groups which cannot consider absolutely unhealthy like fish, fruit juices, coffee and high fat dairy, and therefore this also could be called “mix network”. Nevertheless, the most important part is that all food groups were eaten in relation with central group(s). Therefore, it is better to pay special attention to central food groups in the networks. Cooked vegetables and processed meat were the central food groups of our healthy and unhealthy networks, respectively, which showed no significant association with MetS. This result was also supported by the recent systematic review and dose–response meta-analysis study on the association between consumption of fruit and vegetable and MetS. They have showed that high consumption of vegetables was related with a reduced risk of hyperglycemia but not with Mets [35].

The main strength of the present study is the use of GGM as a novel approach that indicates independent correlations among food groups and helps us to recognize central food groups, which PCAs or simple correlation analyses cannot. Further study strength is use of validated semi-quantitative FFQ as a dietary assessment tool. Additionally, this is the first study which assesses the relation among GGM dietary networks and MetS.

The current study has some limitations. For instance, FFQ could assess the usual intake not actual intake and also depends on the participant’s memory which could cause mistakes over-reporting and under-reporting of intakes. Another limitation is the cross-sectional design of the study which disables us to perceive the cause-and-effect relationship between dietary networks and Mets. Naming the identified dietary networks is also a limitation in comparing results. Additionally, the data need to be Gaussian-distributed in GGM method which is not possible for all variables. We also know that the effect of different kinds of foods on health condition depends on the preparation and cooking methods [36–42]. Besides, there are some other effective confounders like meal time, sleep duration, stress, genetics and etc. which could not adjust in this study.

## Conclusion

In conclusion, the GGM derived dietary networks represents dietary patterns and able to recognize central food groups. This study showed that saturated fats network was associated with chance of having MetS and hyperglycemia. Consequently, additional prospective studies need to validate this method in other populations.

## Data Availability

The data generated or analyzed during the current study are not publicly available but are available from the corresponding author upon reasonable request.
